# Use of Oncotype DX in Women with Node-Positive Breast Cancer

**DOI:** 10.1371/currents.RRN1249

**Published:** 2011-07-21

**Authors:** Naoko Ishibe, Sheri Schully, Andrew Freedman, Scott David Ramsey

**Affiliations:** ^*^Program Director, CTEB, DCCPS, National Cancer Institute, NIH, Bethesda, MD; ^†^Health Sciences Administrator, NCI, Bethesda, MD; ^‡^Genomic Pharmacoepidemiology, US National Cancer Institute and ^§^Physician, Health Services Researcher

## Abstract

Women with early stage breast cancer frequently receive adjuvant chemotherapy to prevent recurrence; however, not all patients benefit. Recently, gene expression marker panels, such as Oncotype DX, that may better predict risk of breast cancer recurrence have become commercially available and are being used to guide treatment decisions. Oncotype DX analyzes the expression of 21 genes within a tumor to determine a recurrence score that corresponds to a specific likelihood of breast cancer recurrence within 10 years of the initial diagnosis, as well as response to adjuvant treatment. We examined the published literature on the analytic validity, clinical validity, and clinical utility of Oncotype DX in guiding adjuvant treatment decisions in women with lymph node-positive breast cancer.

## **Clinical Scenario**

Women with early stage breast cancer frequently receive adjuvant chemotherapy based on standard recurrence risk classification using lymph node status and qualitative tumor characteristics, such as size, type, grade, receptor status, and histology.  These classifiers identify very few women who are at low risk of recurrence and as a result, more patients are treated with chemotherapy than those who will benefit.  Panels of gene expression markers—such as Oncotype DX^TM—^are marketed to physicians and patients for having the benefit of better predicting risk of breast cancer recurrence and to guide treatment decisions in women with lymph node-negative breast cancer.  However, it is not clear whether Oncotype DX can accurately assess risk of disease recurrence in women with lymph node-positive breast cancer. 

**Test Description**

Oncotype DX^TM ^ analyzes the expression of 21 genes (16 cancer-related and 5 normative genes) within a tumor to determine a recurrence score (RS) using reverse transcription PCR in formalin-fixed, paraffin-embedded breast cancer tissue samples. The RS is a number between 0 and 100 that corresponds to a specific likelihood of breast cancer recurrence within 10 years of the initial diagnosis, as well as response to adjuvant treatment. Using recurrence score, it may be possible for healthcare providers and patients to determine whether adjuvant chemotherapy is needed following primary therapy for breast cancer.

**Public Health Importance**

Breast cancer is the most commonly diagnosed cancer in U.S. women and is the second leading cause of cancer-related deaths in 2010 [1].  Although breast cancer patients frequently receive adjuvant chemotherapy to prevent recurrence, not all patients benefit. Gene expression tests may aid in chemotherapy decision-making.  Specifically, women with low RS may have low likelihood of recurrence and thus may choose to avoid the potential toxicity and morbidity of chemotherapy.  

**Published Reviews, Recommendations and Guidelines**

**Systematic evidence reviews/ technology assessments**

      Blue Cross Blue Shield Association, Technology Evaluation Center (BCBS TEC) [2] [3]

**Recommendations by independent group**

      None identified that evaluated the use of Oncotype DX in lymph node-positive breast cancer.

**Guidelines by professional groups**

      None identified.

With the exception of BCBS TEC, none of the professional or independent groups evaluated Oncotype DX for lymph node-positive breast cancer. BCBS TEC concluded that there is insufficient evidence to determine the clinical validity or utility of Oncotype DX as a predictor of breast cancer recurrence or response to adjuvant chemotherapy in patients with node-positive breast cancer.    

**Evidence Overview**

***Analytical Validity*: ** test accuracy and reliability in measuring differences in expression of relevant genes (analytic sensitivity and specificity).

      Based on evaluation by EGAPP [4], because there is no referent technology, it is not possible to estimate the false positive or false negative rates of the test.  Based on seven studies, testing initially failed in 14.5% of samples due to insufficient tumor content, poor RNA samples, and RT-PCR failure.  However, no studies have been conducted in node-positive breast cancer patients.

      Cronin et al. [5] assessed the amplification efficiency, linearity, quantification limitations, dynamic range, analytical precision, and reproducibility performance of Oncotype DX. The authors reported that the reproducibility of the recurrence score was very high with repeat testing of de-identified patient samples (standard deviation of less than 2 recurrence score units). 

***Clinical Validity*: ** test accuracy and reliability in predicting recurrence in node-positive breast cancer patients (prognosis) and benefit from chemotherapy (predictive).

- Retrospective analysis of the phase 3 trial SWOG-8814 reported on the clinical validity of Oncotype DX^TM^ in women who had node-positive breast cancer and were treated with tamoxifen alone [6].  The authors state that the study suggests that patients with node-positive breast cancer with a low recurrence score do not seem to benefit from adjuvant chemotherapy (hazard ratio [HR]=1.02, 95% confidence interval [CI] = 0.54-1.93), whereas those with a high recurrence score show an improvement in disease-free survival, independent of the number of positive nodes (HR=0.59, 95% CI = 0.35-1.01). The RS was also prognostic in the tamoxifen-alone group (HR=2.64, 95% CI – 1.33 -5.27, for a 50-point difference in RS).
- Dowsett et al. [7] evaluated whether the recurrence score based on Oncotype DX provided independent information on risk of distant recurrence in the tamoxifen and anastrozole arms of the ATAC Trial.  The authors reported that recurrence score is an independent prognosticator of recurrence in node-positive (as well as node-negative) breast cancer patients (HR=3.47, 95% CI = 1.64-7.38, for a 50-point difference in RS) and concluded that it provides additional information to risk determined using standard clinicopathologic features. 

***Clinical Utility*: ** net benefit of test in improving health outcomes.

- No prospective studies assessing clinical utility have been conducted in women with node-positive breast cancer.
- It is not clear whether recurrence scores for women with lymph-node positive breast cancer derived by the use of Oncotype DX improves health outcome beyond current standard clinical classification methods [3].
- SWOG S-1007, a prospective, randomized trial to determine the effect of chemotherapy in patients with 1-3 positive nodes, and hormone receptor-positive, HER2-negative breast cancer who do not have high RS by Oncotype DX.  This trial, which is scheduled to be completed in 2016 will provide evidence regarding the clinical utility of Oncotype DX [8].

**Limitations**

      There is currently no data clearly demonstrating clinical utility of Oncotype DX in women with lymph node-positive breast cancer. 

**Conclusions**

Recently, gene expression marker panels that may better predict risk of breast cancer recurrence have become commercially available and are being used to guide treatment decisions in women with node-negative breast cancer. This has led to one prospective, randomized controlled study specifically focused on women with lymph node-positive breast cancer to assess whether those panels can better predict recurrence in those patients as well.  Although there is some evidence to suggest that the use of Oncotype DX can provide additional information in predicting recurrence in women with lymph node-positive breast cancer, there are currently no data from prospective clinical trials assessing the relative clinical benefit of OncotypeDX-guided therapy vs. current care in those women.  Therefore, it is unclear whether results of the gene expression panel can be used to withhold chemotherapy for a portion of women who otherwise would receive it as part of therapy.  Thus, current data cannot answer the question of whether Oncotype Dx-guided practice improves health outcomes beyond standard clinical practice. 

**Links**

Last updated: July 21, 2011

**Acknowledgements**

The authors would like to thank Dr. Muin Khoury of the Centers for Disease Control and Prevention and Dr. David Veenstra of the University of Washington for their invaluable input and guidance on the content.  The authors also acknowledge the contributions of Ms. Camilla Benedicto and Ms. Kelly Bennett of the National Cancer Institute in supporting this project.   

**Funding information**

This study was funded, in part, by CANCERGEN (Comparative Effectiveness Research in Cancer Genomics) through the American Recovery and Reinvestment Act of 2009 by the National Cancer Institute, National Institutes of Health under Agency Award #RC2CA138570 (Principal Investigator: Scott D. Ramsey). 

**Competing interests**

The authors have declared no competing interests exist.

**Disclaimers**

The findings and conclusions are those of the authors and do not necessarily represent the views of the National Institutes of Health (NIH).  The information provided in this manuscript does not constitute an endorsement of Oncotype DX by NIH nor the Department of Health and Human Services of the U.S. government.

****
